# Study on the prediction model of atherosclerotic cardiovascular disease in the rural Xinjiang population based on survival analysis

**DOI:** 10.1186/s12889-023-15630-x

**Published:** 2023-06-01

**Authors:** Xin Qian, Mulatibieke Keerman, Xianghui Zhang, Heng Guo, Jia He, Remina Maimaitijiang, Xinping Wang, Jiaolong Ma, Yu Li, Rulin Ma, Shuxia Guo

**Affiliations:** 1 Department of Public Health, Shihezi University School of Medicine, Suite 721, The Key Laboratory of Preventive Medicine, Building No. 1, Beier Road, ShiheziShihezi, 832000 Xinjiang China; 2 Department of Public Health, The Key Laboratory of Preventive Medicine, Shihezi University School of Medicine, Suite 816Building No. 1, Beier Road, Shihezi, 832000 Xinjiang China; 3 Department of NHC Key Laboratory of Prevention and Treatment of Central, Asia High Incidence Diseases, The First Affiliated Hospital of Shihezi University Medical College, Shihezi, Xinjiang China

**Keywords:** ASCVD, Machine learning, Predictive models, Survival analysis

## Abstract

**Purpose:**

With the increase in aging and cardiovascular risk factors, the morbidity and mortality of atherosclerotic cardiovascular disease (ASCVD), represented by ischemic heart disease and stroke, continue to rise in China. For better prevention and intervention, relevant guidelines recommend using predictive models for early detection of ASCVD high-risk groups. Therefore, this study aims to establish a population ASCVD prediction model in rural areas of Xinjiang using survival analysis.

**Methods:**

Baseline cohort data were collected from September to December 2016 and followed up till June 2022. A total of 7975 residents (4054 males and 3920 females) aged 30–74 years were included in the analysis. The data set was divided according to different genders, and the training and test sets ratio was 7:3 for different genders. A Cox regression, Lasso-Cox regression, and random survival forest (RSF) model were established in the training set. The model parameters were determined by cross-validation and parameter tuning and then verified in the training set. Traditional ASCVD prediction models (Framingham and China-PAR models) were constructed in the test set. Different models' discrimination and calibration degrees were compared to find the optimal prediction model for this population according to different genders and further analyze the risk factors of ASCVD.

**Results:**

After 5.79 years of follow-up, 873 ASCVD events with a cumulative incidence of 10.19% were found (7.57% in men and 14.44% in women). By comparing the discrimination and calibration degrees of each model, the RSF showed the best prediction performance in males and females (male: Area Under Curve (AUC) 0.791 (95%CI 0.767,0.813), C statistic 0.780 (95%CI 0.730,0.829), Brier Score (BS):0.060, female: AUC 0.759 (95%CI 0.734,0.783) C statistic was 0.737 (95%CI 0.702,0.771), BS:0.110). Age, systolic blood pressure (SBP), apolipoprotein B (APOB), Visceral Adiposity Index (VAI), hip circumference (HC), and plasma arteriosclerosis index (AIP) are important predictors of ASCVD in the rural population of Xinjiang.

**Conclusion:**

The performance of the ASCVD prediction model based on the RSF algorithm is better than that based on Cox regression, Lasso-Cox, and the traditional ASCVD prediction model in the rural population of Xinjiang.

**Supplementary Information:**

The online version contains supplementary material available at 10.1186/s12889-023-15630-x.

## Introduction

Atherosclerotic cardiovascular disease (ASCVD), which mostly involves heart attacks and strokes caused by atherosclerosis, is one of the main causes of death worldwide [1]. Related studies have demonstrated that ASCVD exhibits the characteristics of a long incubation period and severe symptoms at diagnosis. However, early intervention has been shown to produce significant preventive and treatment effects [2, 3].

Therefore, domestic and foreign ASCVD prevention guidelines recommend using the ASCVD risk prediction model and early detection of high-risk groups to develop interventions for reducing the risk of ASCVD in the population [4, 5]. Traditional ASCVD prediction models include the American Framingham model (FRS) [6], Pooled Cohorts Equations (PCE) [7], and Prediction for ASCVD Risk in China (China-PAR) model [8]. However, the external validation of different populations demonstrates that the traditional model underestimates or overestimates disease risk to a certain extent [9–11].

Located in northwestern China, Xinjiang is a multi-ethnic gathering area composed of individuals from the Uygur, Han, and Kazakh ethnic groups and other ethnic groups. Previous studies have demonstrated that the prevalence rates of ASCVD risk factors, such as metabolic syndrome, hypertension, and obesity, are high in Uygur and Kazakh people in rural areas of Xinjiang, leading to an increased risk of ASCVD in this population [12–14]. The study also demonstrated that common prediction models were unsuitable for identifying high-risk ASCVD populations [15].

Survival analysis is a statistical analysis method that considers the outcome of an event and the time taken for the occurrence of the result as the observation outcome [16]. Currently, the most commonly used survival analysis method is the Cox proportional hazards model, and most traditional ASCVD prediction models are constructed based on this model. Due to the need for Cox regression to meet the requirements of proportional risk and independence between variables, the number of variables included in the model may be limited. ASCVD is a complex chronic disease caused by multiple risk factors, so using Cox regression to establish a prediction model may not help to predict individual disease risk well. The development of information technology and machine learning algorithms have been applied in the field of survival analysis and play an important role. In addition, there is some controversy regarding prediction models used for survival analysis. Some studies believe that the prediction model based on a machine learning algorithm is better than the Cox regression model [17–19]. Still, other studies have demonstrated that the prediction performance of the Cox regression model is not lower than that of the machine learning algorithm [20, 21]. In addition, the current comparisons between traditional models and machine learning algorithms in cardiovascular disease prediction models are mostly based on logistic regression, random forest, support vector machine, and other algorithms to build models for comparison. At the same time, few use survival analysis methods for the model construction and comparison [22–24].

Therefore, this study used the Uygur population of Xinjiang as an example to establish a prediction model based on survival analysis suitable for ASCVD risk, early identification of high-risk groups for ASCVD, and provide a theoretical basis for the effective prevention of ASCVD in the future in rural areas of Xinjiang. This study has great practical significance for the comprehensive prevention and control of ASCVD in the communities in this region.

## Methods

### Study population

**Fig. 1:**
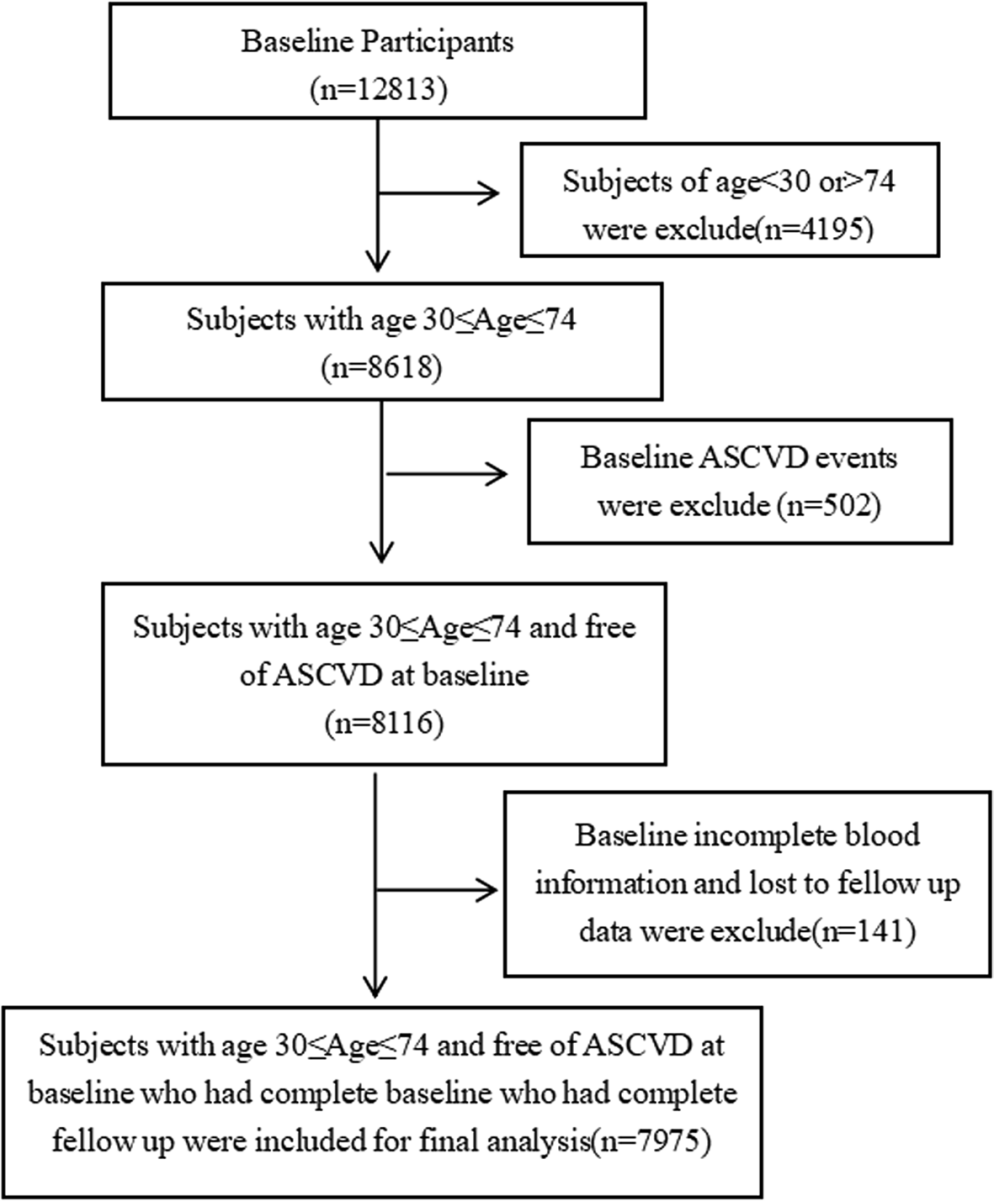
Flow chart of inclusion and exclusion of cohort population ASCVD,Atherosclerotic cardiovascular disease

This study was conducted in the rural areas of Xinjiang. Through multistage stratified cluster random sampling, the 51st Regiment of the Third Division of Xinjiang Corps was selected as the research object, and baseline information was collected from September 2016 to December 2016. A total of 12,813 people aged ≥ 18 years who had lived in the local area for more than 6 months were included in this study. The median time of follow-up, which lasted until June 2022, was 5.79 years. After excluding those aged < 30 years and those aged > 74 years (*n* = 4195), the patients with ASCVD at baseline (*n* = 502), those lost to follow-up, and those with incomplete information (*n* = 141), 7975 people were included in the final cohort (see Fig. 1 for details). All of the participants provided written informed consent. This study was approved by the ethics committee of the First Affiliated Hospital of Shihezi University School of Medicine (SHZ2010LL01).

### Data collection

Data were collected using questionnaires, physical examinations, and laboratory tests. The questionnaires were administered face-to-face. Trained professionals measured anthropometric data, such as height, weight, waist circumference (WC), HC, blood pressure, and B-ultrasound. For each participant, blood pressure and heart rate were measured three times using a mercury sphygmomanometer after a 5-min sitting rest, and the mean values were calculated. Hypertension was defined as a systolic blood pressure (SBP) of ≥ 140 mmHg or a diastolic blood pressure of (DBP) ≥ 90 mmHg. Prehypertension was defined as 140 > SBP ≥ 120 mmHg or 90 > DBP ≥ 80 mmHg [25]. The composite index was calculated from anthropometric measurements: body mass index (BMI) (weight [kg] / height ^2^ [m]), body obesity index (BAI) (HC / height ^1.5^—18), pulse pressure difference (SBP—DBP), and waist-to-hip ratio (WHR) (WC [cm] / HC [cm]). A family history of diabetes was defined as a history of diabetes in at least one parent or sibling; the same criteria were used for a family history of ASCVD. Current smokers were defined as participants who had smoked for more than 6 months [26]. Alcohol consumption was defined as consuming alcoholic beverages (beer, red wine, or white wine) two times or more per month [27]. Fasting blood samples (5 mL) were collected from each subject, and fasting blood glucose (FBG), triglyceride (TG), high-density lipoprotein cholesterol (HDL-C), total cholesterol (TC), low-density lipoprotein cholesterol (LDL-C), and other indicators were measured at the First Affiliated Hospital of Shihezi University School of Medicine using an automatic biochemical analyzer (Olympus AU 2700; Olympus Diagnostics, Hamburg, Germany). In this study, diabetes [28] was defined as an FBG level of ≥ 7.0 mmol/L, a 2-h postprandial blood glucose level of ≥ 11.1 mmol/L, a previous diagnosis of diabetes, or the use of glycemic control medications. Fatty liver was defined as abdominal B-ultrasound results with two of the following three conditions: 1: diffuse enhancement of near-field echo of the liver or "bright liver," the echo is stronger than that of the kidney; 2: the unclear display of intrahepatic pipeline structure; 3: gradual attenuation of echo in the far field of the liver [29]. We also calculated other composite indexes, including the triglyceride blood glucose index (TyG) (TG [mg/dL] × FBG [mg/dL]), fat accumulation product index (LAP) (men: [WC—65] × TC [mmol/L]; women: [WC—58] × TG [mmol/L]), lipoprotein binding index (LCI) (TC × TG [mmol/L] × LDL-C / HDL-C), atherosclerosis index (AI) (TC [mmol/L]—HDL-C) / HDL-C), AIP (log[TG / HDL]), low–high-density lipoprotein ratio (LpH) (LDL-C / HDL-C), and bilirubin composite index (THT) (TC [mmol/L] / [HDL-C + TBIL (μmol/mL)]).

### Diagnostic criteria

In this study, hypertension [25], diabetes [27], and fatty liver [29] were diagnosed according to the diagnostic criteria of the corresponding guidelines. ASCVD was diagnosed according to the China-PAR study [8] as nonfatal acute myocardial infarction, death from coronary heart disease, or fatal or nonfatal stroke during follow-up. ASCVD outcome events were recorded based on responses to the patient questionnaire and history of hospitalization. If the same ASCVD event occurred more than once, the first ASCVD event was considered the outcome event, and the onset time was recorded. Self-reported patients were required to provide evidence for their clinical diagnoses.

### Forecast model introduction

The Cox proportional hazards regression model, also known as Cox regression, is the most widely used traditional modeling method for survival analysis [30]. The application of this model needs to meet the conditions of equal proportional risk and the absence of a nonlinear effect between the independent variables. Lasso–Cox regression introduces Lasso regression to screen for variables based on Cox regression. By introducing a penalty coefficient into the regression model, the regression coefficient of the less important variable is reduced to zero to reduce the model's complexity and avoid overfitting [31]. RSF is an ensemble-learning algorithm based on a binary survival tree. The binary survival tree is different from the traditional decision tree in that when the data are divided into nodes, the data will be grouped according to the standard of the maximum difference in the survival situation [32]. RSF uses the bootstrap method to randomly select a certain number of samples by returning the data. In each sampling process, 37% of the original data are excluded, constituting out-of-pocket data. The model's predictive performance is evaluated by calculating the error of the out-of-pocket data. The China-PAR and FRS models are established based on Cox regression, a widely recognized ASCVD prediction model. The calculation process of China PAR and FRS models is detailed in Supplementary Table 1.

### Dataset partitioning and variable selection

First, the datasets were divided into a dataset of men and a dataset of women according to the different genders. Each dataset was randomly divided into a training set and a test set at a ratio of 7:3. The K-S test was conducted on the training and testing sets of different genders. The *P* values were greater than 0.05, indicating that the data were evenly distributed in the training and testing sets. The database contains 61 variables, including demographic characteristics, questionnaire information, and serological indicators. If all variables are used in model construction, the computational burden will increase, and the model will easily lead to overfitting, affecting the final predictive performance. Therefore, different screening methods were used for the variable screening. Selecting meaningful features through different variable screening techniques can effectively reduce generalization errors. The methods commonly used in machine learning include regularization, feature selection, and feature extraction based on the RSF algorithm. L1 regularization satisfies the sparsity of weights, that is, the weight of most feature vectors is 0, and the sparsity of weights can reduce the complexity of the model. The RSF algorithm can measure the importance of features by the average impure decay of all survival trees and then rank the features according to their importance, selecting the top-ranked features to build a machine-learning model. The traditional model mainly selects variables by single and multiple factors of Cox regression. This study will use L1 regularized Cox regression, RSF algorithm, and Cox regression analysis to select meaningful characteristic variables.

### Model establishment and verification

Using different subsets of variables, Cox regression, Lasso Cox, and RSF models were constructed in the training set, and parameters were selected through cross-validation to determine the final model parameters. Furthermore, we compared the discrimination and calibration degrees of the China-PAR and FRS models with the model constructed in this study in the test set to determine the most suitable prediction model for this population (see Supplementary Fig. 1 for details). The discrimination of the model was evaluated by comparing AUC, the consistency index (C-statistic) [33], the net reclassification index (cNRI), and the comprehensive discriminant improvement index (integrated discrimination improvement, IDI) [34]. This is determined by calculating the BS (the closer the Brier Score is to 0, the better the calibration is) or Homser–Lemeshow χ^2^ statistic (χ ^2^ > 20 or *P* > 0.05 is considered a good calibration) [35, 36] for judgment. To avoid model overfitting, we used five-fold cross-validation to optimize the parameters of the training set and subsequently selected the optimal model.

### Data analysis

There are some missing values in the database, and directly deleting the missing values will lead to a loss of sample information. As there are a few missing variable values in this study, the mean value is used to fill continuous variables, and the mode is used to fill categorical variables. The continuous variables are expressed as the mean ± standard deviation, categorical variables are expressed as the pass rate and constituent ratio, and differences between the measurement data and count data were analyzed by the *t*-test and chi-square test, respectively. The statistical analyses were performed using SPSS, version 26.0, or R language 4.0, and all data were analyzed separately for men and women. A two-tailed *P* < 0.05 was used to indicate statistically significant differences.

## Results

### Baseline information description

A total of 7975 people were included in this study (4054 men, 3920 women). During a median follow-up of 5.79 years, 873 ASCVD events were observed, with a cumulative incidence of 10.19% (7.57% in men and 14.44% in women). After the Kaplan–Meier curve adjustment was performed, the 5-year ASCVD incidence observed in women was higher than in men. The baseline characteristics are shown in Supplementary Table 2. The women had a higher globulin level, higher heart rate, higher platelet values, higher prevalence of hypertension, higher prevalence of obesity, and higher prevalence of family histories of diabetes and ASCVD than the men. Still, the other indicators were lower in women than men. In addition to waist circumference, SBP, and the proportion of diabetes mellitus, the distribution of other predictive variables between men and women was statistically significant (*P* < 0.05), suggesting that there were gender differences in various risk factors, and risk prediction models should be constructed according to gender.

### Model construction and performance comparison

**Fig. 2:**
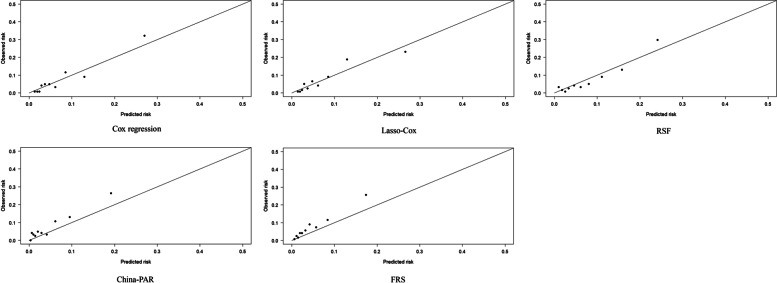
Calibration of different prediction model in male population

**Fig. 3:**
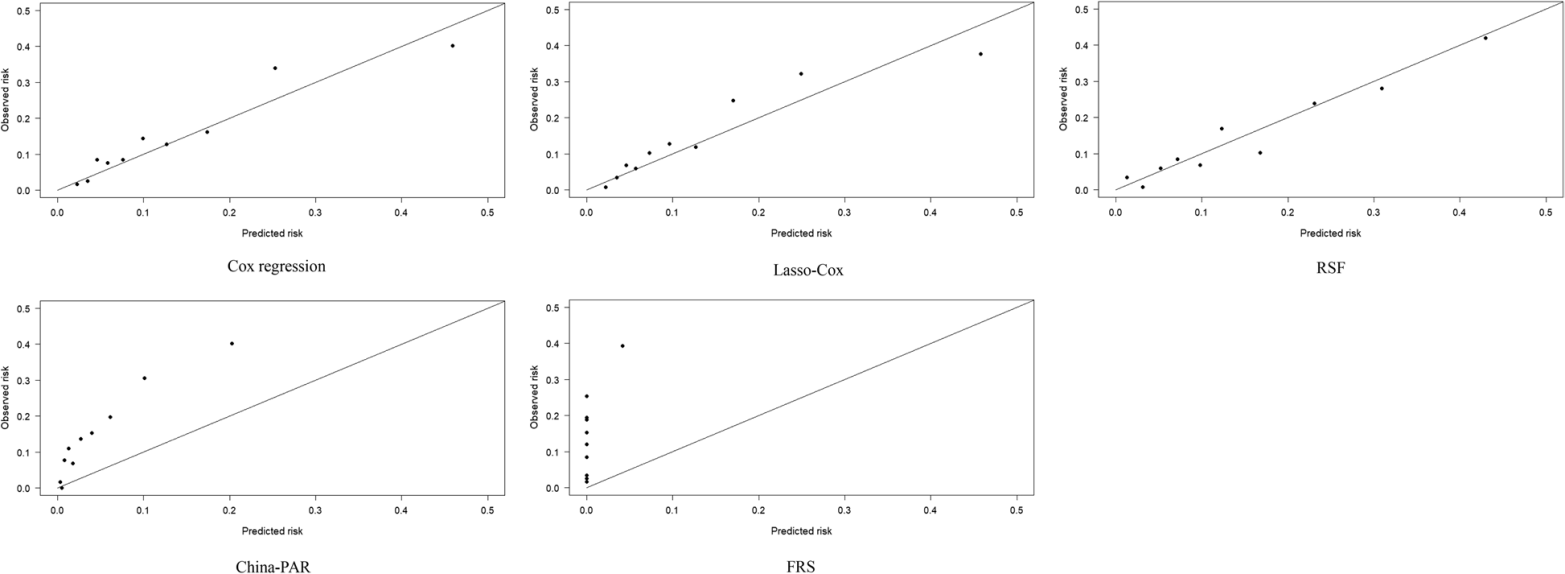
Calibration of different prediction model in female population

We used Cox multivariate analysis, Lasso regression, and RSF to rank the importance of permutation variables in the different gender databases to select the predictor variables. We constructed a prediction model based on the selected variables. Detailed information on the variables is provided in Supplementary Tables 3.1–3.6. To further explore the model's predictive performance, the Cox regression, Lasso–Cox, and RSF models were applied to the database of men and women. The training set was used for cross-validation and parameter tuning to determine the model parameters. The model's discrimination and calibration were further tested in the test set and compared with the traditional China-PAR and FRS models. The results demonstrate no risk of overfitting in each model by comparing the C-statistics of the different models in the training and test sets (see Supplementary Table 4). The predictive performance indices for each model are listed in Table 1. The comparison of the AUC, the C-statistics, Homser–Lemeshow test results, BS model results, Cox regression model results, Lasso–Cox model results, and RSF model results demonstrated moderate discrimination in different gender datasets (AUC, men: 0.729–0.791, women: 0.742–0.759, C-statistic, men: 0.775–0.780, women: 0.733–0.737) and calibration (Homser–Lemeshow test, *P* > 0.05). The discrimination between the China-PAR and FRS models in this population was moderate (AUC, men: 0.729–0.757, women: 0.742–0.756, C statistic, men: 0.738–0.748, women: 0.721–0.734), but the calibration was poor (Homser–Lemeshow test, *P* < 0.05), as shown in Table 1 and Figs. 2 and 3. When comparing the differences in cNRI and IDI between the constructed and traditional models in the rural population of Xinjiang, it was demonstrated that Cox regression, Lasso–Cox, and RSF models performed better than the traditional model of net reclassification ability and comprehensive discrimination ability. Taking the results of China-PAR and RSF models in the population of men as an example, the cNRI value was 0.297 (95% CI: 0.177, 0.427), and the IDI value was 0.050 (95% CI: 0.035, 0.065). Compared with the China-PAR model, the correct classification ability of the RSF model was 29.7% higher, and the comprehensive discriminative ability of the RSF model was 5.0% higher. Similar results were observed in the women. The results of the BS also demonstrated that, in this population, the calibration degree of the prediction model constructed based on Cox regression, Lasso–Cox, and RSF models in this study were better than that of the traditional model, and the RSF prediction model had the best performance, as shown in Tables 2 and 3.

**Table 1:** Comparing discrimination and calibration of different models

	Cox regression	Lasso-Cox	RSF	China-PAR	FRS
Man					
AUC	0.789	0.787	0.791	0.757	0.729
95% CI for the AUC	(0.765–0.812)	(0.763,0.810)	(0.767,0.813)	(0.732,0.781)	(0.723,0.773)
C statistic	0.779	0.775	0.780	0.748	0.738
95% CI for the C statistic	(0.736–0.822)	(0.730,0.819)	(0.730,0.829)	(0.695,0.800)	(0.688,0.789)
Brier Score	0.061	0.062	0.060	0.063	0.064
χ^2^ for Homser-Lemeshow test	10.16	4.20	10.12	49.20	24.35
*P* for Homser-Lemeshow test	0.25	0.84	0.26	< 0.01	< 0.01
Woman					
AUC	0.756	0.759	0.759	0.756	0.742
95% CI for the AUC	(0.730,0.780)	(0.733,0.783)	(0.734,0.783)	(0.730,0.780)	(0.716,0.767)
C statistic	0.733	0.736	0.737	0.734	0.721
95% CI for the C statistic	(0.697,0.768)	(0.701,0.771)	(0.702,0.771)	(0.699,0.768)	(0.686,0.757)
Brier Score	0.111	0.111	0.110	0.125	0.143
χ^2^ for Homser-Lemeshow test	10.84	15.22	3.72	394.87	-
*P* for Homser-Lemeshow test	0.21	0.06	0.88	< 0.01	-

*Abbreviations*: *RSF* random survival fores, *China-PAR* Prediction for ASCVD Risk in China, *FRS* Framingham model

**Table 2:** Comparison of discrimination performance of prediction models in man

Predictive model	cNRI	*P* Value	IDI	*P* Value
Cox regression vs.Lasso-Cox	0.010(-0.199,0.219)	0.93	-0.004(-0.010,0.002)	0.16
Cox regression vs.RSF	0.272(0.063,0.481)	0.01	0.017(0.001,0.034)	0.04
Cox regression vs.China-PAR	0.065(-0.098,0.227)	0.44	-0.033(-0.049,-0.017)	< 0.01
Cox regression vs.FRS	-0.169(-0.297,-0.041)	< 0.01	-0.045(-0.061,-0.029)	< 0.01
Lasso-Cox vs.RSF	0.323(0.128,0.530)	< 0.01	0.021(0.006,0.037)	< 0.01
Lasso-Cox vs.China-PAR	0.094(-0.072,0.260)	0.27	-0.029(-0.044,-0.014)	< 0.01
Lasso-Cox vs.FRS	-0.188(-0.310,-0.067)	< 0.01	-0.041(-0.056,-0.026)	< 0.01
RSF vs.China-PAR	-0.297(-0.427,-0.177)	< 0.01	-0.050(-0.065,-0.035)	< 0.01
RSF vs.FRS	-0.447(-0.564,-0.329)	< 0.01	-0.062(-0.080,-0.045)	< 0.01
China-PAR vs.FRS	-0.381(-0.596,-0.165)	< 0.01	-0.013(-0.020,-0.004)	< 0.01

*Abbreviations*: *cNRI* continuous Net Reclassification Index, *IDI* Integrated Discrimination Improvement Index

**Table 3:** Comparison of discrimination performance of prediction models in woman

Predictive model	cNRI	*P* Value	IDI	*P* Value
Cox regression vs.Lasso-Cox	-0.144(-0.305,0.018)	0.08	-0.002(-0.008,0.004)	0.56
Cox regression vs.RSF	-0.016(-0.174,0.141)	0.84	-0.011(-0.030,0.009)	0.29
Cox regression vs.China-PAR	0.024(-0.028,0.077)	0.37	-0.078(-0.099,-0.056)	< 0.01
Cox regression vs.FRS	-0.012(-0.022,-0.002)	< 0.01	-0.125(-0.153,-0.096)	< 0.01
Lasso-Cox vs.RSF	-0.032(-0.190,0.126)	0.69	-0.009(-0.028,0.010)	0.36
Lasso-Cox vs.China-PAR	0.005(-0.044,0.053)	0.85	-0.076(-0.096,-0.055)	< 0.01
Lasso-Cox vs.FRS	-0.010(-0.019,-0.001)	0.02	-0.123(-0.151,-0.095)	< 0.01
RSF vs.China-PAR	0.008(-0.016,0.031)	0.52	-0.067(-0.081,-0.053)	< 0.01
RSF vs.FRM	-	-	-0.114(-0.135,-0.093)	< 0.01
China-PAR vs.FRS	-0.004(-0.010,-0.002)	0.16	-0.047(-0.060,-0.035)	< 0.01

*Abbreviations*: *cNRI* continuous Net Reclassification Index, *IDI* Integrated Discrimination Improvement Index

In the population of men, the AUC and C-statistics of the Cox regression, Lasso–Cox, and RSF models were similar to and higher than those of the China-PAR and FRS models, respectively. Further comparisons of the cNRI and IDI between the Cox regression, Lasso–Cox, and RSF models and the net reclassification ability and comprehensive discrimination ability between the RSF, Cox regression, and Lasso–Cox models demonstrated statistically significant differences. Taking the comparison between Lasso–Cox and RSF as an example, the cNRI value was 0.323 (95% CI: 0.128, 0.530), and the IDI value was 0.021 (95% CI: 0.006, 0.037), indicating that the predictive ability of Lasso–Cox model was higher than that of the RSF model. Correct classification and comprehensive discrimination abilities improved by 32.3% and 2.1%, respectively. The calibration curves demonstrate that the three models had good calibration (Homser–Lemeshow: χ^2^ > 20, *P* > 0.05). In the population of women, the AUC and C-statistics of the Lasso–Cox, RSF, and China-PAR models were similar to and higher than those of the Cox regression and FRS models, respectively. The cNRI and IDI of the Cox regression, Lasso–Cox, and RSF models were compared, and the differences between the three models were not statistically significant. The calibration curve results demonstrate that the numbers of patients with ASCVD predicted by the Cox regression, Lasso–Cox, and RSF models in the population of women were 156.68, 155.84, and 179.68, respectively. The corresponding ASCVD events/objective ASCVD events predicted by the Cox regression, Lasso–Cox, and RSF models were 0.92, 0.91, and 1.04, respectively.

### Importance of ranking of variables in different models

**Fig. 4:**
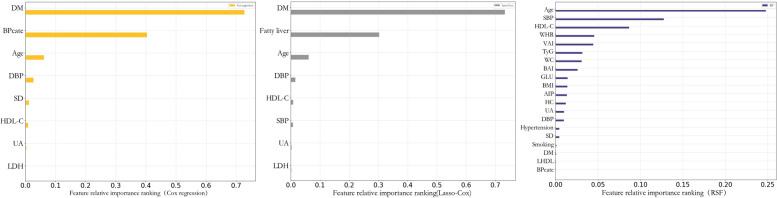
Importance of Cox regression, Lasso-Cox and RSF variables in male population

**Fig. 5:**
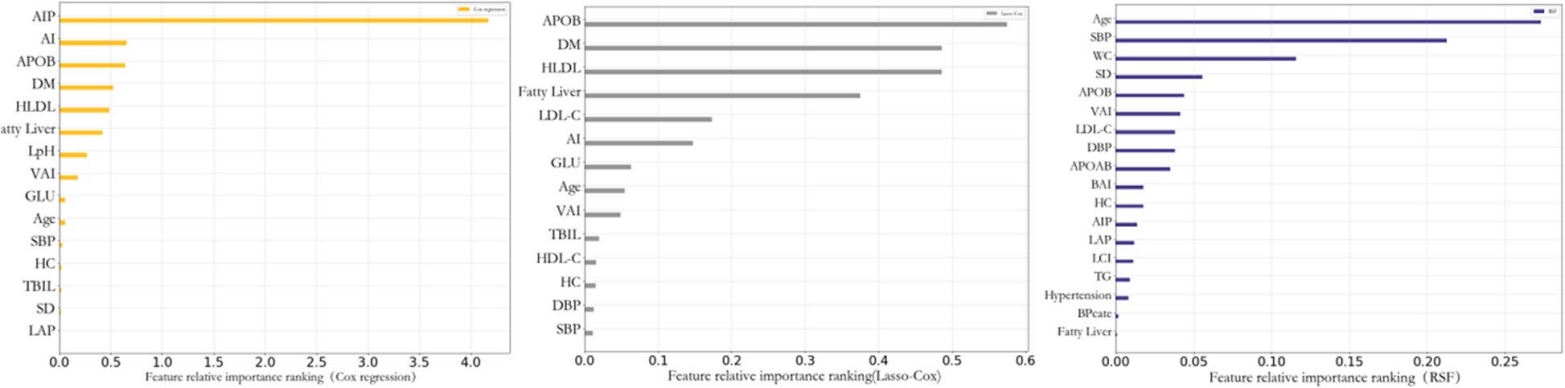
Importance of Cox regression, Lasso-Cox and RSF variables in female population

The importance of the variables in each prediction model was output according to sex to compare the ability of each variable to predict the incidence of ASCVD. In addition to the traditional risk factors for ASCVD, such as age, blood pressure, and diabetes, metabolic indicators, such as lactate dehydrogenase (LDH) and uric acid (UA), VAI, BAI, and WHR, which reflect the degree of obesity, were also important predictors of ASCVD in men. In the population of women, in addition to traditional risk factors such as age and systolic blood pressure, HC, VAI, and LAP reflecting human obesity and lipid metabolism indicators such as APOB and AIP are also important predictors of ASCVD, as shown in Figs. 4 and 5.

## Discussion

The results of this study demonstrate that the cumulative incidence of ASCVD in populations in rural Xinjiang is 10.19%, which is a cumulative incidence of ASCVD that is similar to that in European and American Caucasians [37] and that is higher than that of the Han population [38–40], which may be related to the genetic background and unique dietary habits of this population. Currently, there are few studies on ASCVD in this population. Therefore, this study used survival analysis to establish a prediction model and explore this population's main risk factors for ASCVD.

The predicted incidence of ASCVD was higher in women than in men. A pilot study demonstrated that the prevalence of diseases, such as obesity, metabolic syndrome, and hypertension, is higher in women than men [14, 41]. Relevant research demonstrates that the population of women of a menopausal age experience amalgamations earlier, but due to a lack of estrogen to protect postmenopausal women, ASCVD risk will increase significantly and gradually compared with men [42, 43]. Additionally, this population generally has a large number of children [44] and endocrine disorders caused by various complications during pregnancy, and the lack of local medical resources [45] also increases the risk of ASCVD, to a certain extent, in women of this population.

The comparison of ASCVD risk models in this population demonstrated that the discrimination of the RSF model in the population of men was similar to that of the Lasso–Cox and Cox regression models and higher than that of the China-PAR and FRS models. The comparison between the cNRI and IDI demonstrated that RSF was better than the other models. The results indicated that the RSF model could accurately distinguish between the population of men with ASCVD and the population of men without ASCVD. The calibration results demonstrate that all models have good calibration, and the results of the BS demonstrate that the RSF model is slightly better than those of the other models. In the population of women, the discrimination of all models demonstrated that AUC and the C-statistics of all models were similar and that there was no difference between the cNRI and IDI of the RSF, Lasso–Cox, and Cox regression models. Still, they were all higher than those in the China-PAR and FRS models. The calibration analysis results demonstrated that the performance of the RSF model in this population of women was better than that of the other models. Based on the discrimination and calibration results, the RSF model had the best predictive performance in this population.

The RSF model is an extension of random forest in survival analysis. Unlike the traditional Cox regression model, which needs to satisfy many assumptions, the RSF model is completely nonparametric, does not require restrictive assumptions, and can automatically evaluate the influence of all variables. Therefore, it is widely used in constructing prognostic models for heart failure, arrhythmia, multiple myeloma, and other diseases [43, 46, 47]. In this study, the RSF model demonstrated a better predictive performance than the traditional Cox regression model and Lasso–Cox model for both the men and women in this population, which is a finding that is similar to the findings of the study by Zhang et al. [37–39]. This may be related to the characteristics of the RSF model, which has good processing of complex and high-dimensional data. Some research results show that the Cox model is susceptible to the influence of variable dimensions and equal proportional risks. When the data do not meet the equal proportional assumption or the dimension changes, the robustness of the RSF model is better than that of the Cox model [40, 48].

A comprehensive analysis of the importance of variables in different gender prediction models demonstrated that the most important predictors of ASCVD in men were age and high-density lipoprotein cholesterol, which is a finding that is consistent with the conclusions of many previous studies [6, 8]. APOB was a significant predictor in women. Because each atherogenic particle contains an APOB molecule, the detected concentration of APOB can be considered clinically as the amount of ASCVD lipoprotein [49]. Moreover, a meta-analysis demonstrated that the risk of ASCVD could be reduced by 39% by reducing the concentration of APOB to that of the target level [50]. Studies have demonstrated that the menopausal age of women in this population is earlier than that of women in the Han population, and the level of APOB is affected by estrogen. Therefore, compared with women in other populations, the level of APOB in women in this population is an important factor influencing ASCVD risk prediction. In addition, metabolic indicators, such as AIP and AI, and indicators reflecting body obesity, such as VAI and HC, exhibited a strong ability to predict ASCVD in this population, which may be related to the high prevalence rates of obesity and dyslipidemia in this population caused by high-salt and high-fat diets.

Although we believe that the included population represents the general Uygur population, this study has some limitations. First, only the traditional risk factors were considered. With the deepening of omics research, a large amount of data were entered into the clinical prediction model research. The relevant data based on genomics and metabolomics were not included in this study, which may have a certain impact on the model's predictive performance. Second, this study lacked an independent, external validation population. Although we divided the training and test sets and conducted cross-validation in the training set, the predictive accuracy and robustness of the established model extrapolated to other ethnic populations need to be explored further. Finally, only baseline measurements were used for modeling in this study, and the time effect was not considered in the model construction process. Subsequent studies should incorporate relevant omics data and multiple follow-up data for the modeling analysis and validation in independent external populations.

## Conclusion

In this study, the performance of the ASCVD prediction model based on the RSF algorithm was better than that of those based on Cox regression, Lasso–Cox, and the traditional ASCVD prediction model in the rural population of Xinjiang. In addition to the traditional risk factors for predicting ASCVD, lipid metabolism indicators, such as APOB, AI, and AIP, and obesity indicators, such as BMI and BAI, are considered important factors for predicting the incidence of ASCVD in this population.

## Supplementary Information

**Additional file 1:Supplementary Fig. 1.** Flow chart of data analysis in this study. **Supplementary Table 1. **Parameters of two risk equations used in this study for men and women. **Supplementary Table 2. **Comparison of research objects between man and woman. **Supplementary Table 3.1. **Screening variable subsets based on Cox regression in man. **Supplementary Table 3.2. **Screening variable subsets based on Lasso-Cox regression in man. **Supplementary Table 3.3. **Screening variable subsets based on RSF in man. **Supplementary Table 3.4. **Screening variable subsets based on Cox regression in woman. **Supplementary Table 3.5. **Screening variable subsets based on Lasso-Cox regression in woman. **Supplementary Table 3.6. **Screening variable subsets based on RSF in woman. **Supplementary Table 4. **C statistic of different models on training and test sets.

## Data Availability

The datasets used during the current study are available from the corresponding author on reasonable request. The Chinese questionnaire copy may be requested from the authors.
