# Supplementary material for: Study on the prediction model of atherosclerotic cardiovascular disease in the rural Xinjiang population based on survival analysis
Source: BMC Public Health. 2023 Jun 1;23:1041. doi: 10.1186/s12889-023-15630-x (PMC10234013; doi:10.1186/s12889-023-15630-x)
Supplement: Supplementary file 1 — Additional file 1:Supplementary Fig. 1. Flow chart of data analysis in this study. Supplementary Table 1. Parameters of two risk equations used in this study for men and women. Supplementary Table 2. Comparison of research objects between man and woman. Supplementary Table 3.1. Screening variable subsets based on Cox regression in man. Supplementary Table 3.2. Screening variable subsets based on Lasso-Cox regression in man. Supplementary Table 3.3. Screening variable subsets based on RSF in man. Supplementary Table 3.4. Screening variable subsets based on Cox regression in woman. Supplementary Table 3.5. Screening variable subsets based on Lasso-Cox regression in woman. Supplementary Table 3.6. Screening variable subsets based on RSF in woman. Supplementary Table 4. C statistic of different models on training and test sets. [file 12889_2023_15630_MOESM1_ESM.docx]

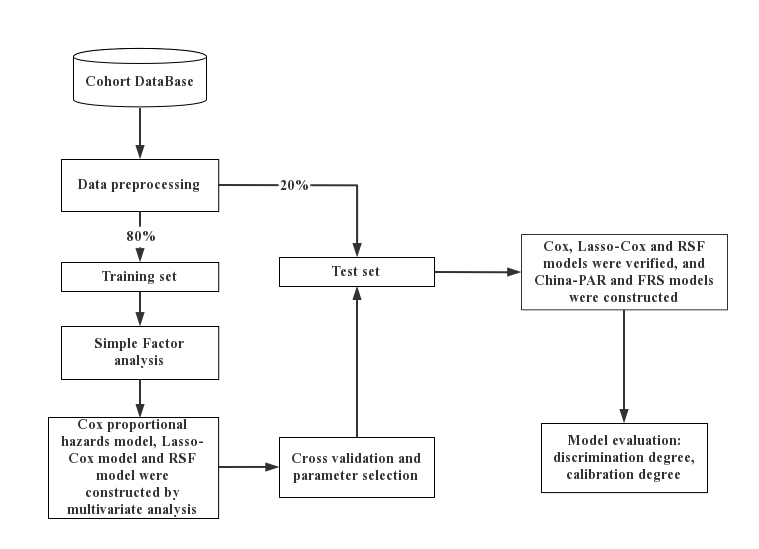


**Supplementary：Fig. 1** Flow chart of data analysis in this study

| **Supplementary：Table 1. Parameters of two risk equations used in this study for men and women** | | |
| --- | --- | --- |
| **Model** | **Equations for PAR and FRS** | |
| **Men** | |  |
| PAR | =31.97×ln(age)+0.62×ln(TC)-0.69×ln(HDLC)-0.71×ln(waist) (+[27.39-6.02×ln(age)]×ln(SBP) if hypertension treated) (+[26.15-5.73×ln(age)]×ln(SBP) if hypertension untreated) (+3.96-0.94×ln(age) if current smoker) (+6.22-1.53×ln(age) if having family history of ASCVD) (+0.36 if diabetes) (+0.48 if in Northern China) (-0.16 if living in urban) | |
| FRS | =3.0617×ln(age)+1.12370×ln(TC)-0.93263×ln(HDLC) (+1.93303×ln(SBP) if hypertension untreated) (+1.99881×ln(SBP) if hypertension treated) (+0.65451 if current smoker) (+0.57367 if diabetes) | |
| **Women** | |  |
| PAR | =24.87×ln(age)+0.06×ln(TC)-0.22×ln(HDLC)+1.48×ln(waist) (+[20.71-4.53×ln(age)] ×ln(SBP) if hypertension treated) (+[19.98 -4.36×ln(age)]×ln(SBP) if hypertension untreated) (+0.49 if current smoker) (+0.57 if diabetes) (+0.54 if in Northern China) | |
| FRS | =2.32888×ln(age)+1.20904×ln(TC)-0.70833×ln(HDLC) (+1.76157×ln(SBP) if hypertension untreated) (+2.82263×ln(SBP) if hypertension treated) (+0.52873 if current smoker) (+0.69154 if diabetes) | |

| **Supplementary：Table 2** Comparison of research objects between man and woman | | | | | |
| --- | --- | --- | --- | --- | --- |
| Feature |  | Man  (n=4054) | Woman  (n=3920) | t/χ^2^ | *P* value |
| Age(years) |  | 44.08±10.85 | 43.31±10.38 | 3.25 | <0.01 |
| Height(cm) |  | 167.57±7.25 | 157.57±6.75 | 63.69 | <0.01 |
| Weight(Kg) |  | 75.30±12.61 | 67.89±12.36 | 26.48 | <0.01 |
| WC(cm) |  | 95.48±13.11 | 93.32±14.06 | 7.11 | <0.01 |
| HC(cm) |  | 102.31±8.90 | 102.72±10.01 | -1.91 | 0.06 |
| FBG (mmol/L) |  | 5.21±2.51 | 5.00±2.05 | 4.07 | <0.01 |
| TG (mmol/L) |  | 2.01±1.55 | 1.73±1.34 | 8.48 | <0.01 |
| TC (mg/dL) |  | 188.50±53.27 | 185.47±54.24 | 2.52 | 0.01 |
| HDL-C(mg/dL) |  | 56.75±22.02 | 59.80±22.82 | -6.08 | <0.01 |
| LDL-C(mmol/L) |  | 2.75±0.90 | 2.69±0.93 | 3.14 | <0.01 |
| SBP (mmHg) |  | 132.15±19.84 | 131.22±21.61 | 2.02 | 0.04 |
| DBP (mmHg) |  | 76.38±12.68 | 76.56±12.72 | -0.63 | 0.53 |
| ALT (mmol/L) |  | 34.25±22.25 | 27.70±19.20 | 14.07 | <0.01 |
| AST (mmol/L) |  | 25.99±12.39 | 23.74±11.01 | 8.53 | <0.01 |
| ALB(g/L) |  | 46.02±3.68 | 44.43±3.75 | 19.07 | <0.01 |
| GLO(g/L) |  | 29.67±5.27 | 30.28±4.98 | -5.36 | <0.01 |
| TBIL(umol/L) |  | 11.77±6.17 | 10.54±5.82 | 9.12 | <0.01 |
| DBIL(umol/L) |  | 4.74±3.14 | 4.23±3.19 | 7.16 | <0.01 |
| ALP(U/L) |  | 78.26±22.71 | 71.50±24.86 | 12.69 | <0.01 |
| GGT(U/L) |  | 24.32±20.13 | 19.61±18.46 | 10.89 | <0.01 |
| CR(umol/L) |  | 77.28±15.19 | 65.59±13.99 | 35.71 | <0.01 |
| AOPAB(g/L) |  | 1.17±0.45 | 1.34±0.45 | -16.57 | <0.01 |
| AOPB(g/L) |  | 0.98±0.25 | 0.92±0.25 | 9.67 | <0.01 |
| HBDH |  | 161.64±64.14 | 158.32±54.35 | 2.49 | 0.01 |
| LDH |  | 181.53±84.36 | 177.73±70.88 | 2.18 | 0.03 |
| UA |  | 286.24±72.11 | 229.63±65.93 | 36.56 | <0.01 |
| Hemoglobin |  | 156.41±18.13 | 140.51±20.47 | 36.76 | <0.01 |
| Leukocyte |  | 7.19±2.24 | 6.87±1.80 | 6.85 | <0.01 |
| Platelets |  | 286.63±66.65 | 306.90±71.41 | -13.11 | <0.01 |
| Heart rate |  | 79.08±10.79 | 81.19±11.14 | -8.58 | <0.01 |
| CKMB |  | 19.64±20.59 | 17.33±17.67 | 5.35 | <0.01 |
| SD(mmHg) |  | 55.78±15.56 | 54.66±16.30 | 3.14 | <0.01 |
| WHR |  | 0.93±0.11 | 0.91±0.11 | 10.29 | <0.01 |
| BMI(Kg/m^2^) |  | 26.86±4.67 | 27.40±5.10 | -4.90 | <0.01 |
| TyG |  | 5.65±7.93 | 4.62±5.78 | 6.60 | <0.01 |
| LAP |  | 151.59±86.18 | 64.81±63.51 | 51.05 | <0.01 |
| BAI |  | 29.32±5.08 | 34.10±6.00 | -38.48 | <0.01 |
| VAI |  | 2.10±2.07 | 2.52±2.50 | -8.13 | <0.01 |
| LCI |  | 22.43±27.67 | 17.72±24.32 | 8.05 | <0.01 |
| AI |  | 2.63±1.52 | 2.39±1.88 | 6.20 | <0.01 |
| AIP |  | 0.53±0.17 | 0.50±0.16 | 8.39 | <0.01 |
| LpH |  | 2.08±0.89 | 1.93±1.06 | 7.00 | <0.01 |
| THT |  | 0.48±0.33 | 0.52±0.31 | -5.80 | <0.01 |
| ASCVD,n(%) | No | 3748(92.43) | 3354(85.56) | 4882.13 | <0.01 |
|  | Yes | 307(7.57) | 566(14.44) |  |  |
| FhDM,n(%) | No | 3840(94.7) | 3644(93.0) | 10.43 | <0.01 |
|  | Yes | 215(5.3) | 276(7.0) |  |  |
| FhASCVD,n(%) | No | 3768(92.9) | 3545(90.4) | 16.22 | <0.01 |
|  | Yes | 287(7.1) | 375(9.6) |  |  |
| DM,n(%) | No | 3732(92.0) | 3626(92.5) | 0.61 | 0.44 |
|  | Yes | 323(8.0) | 294(7.5) |  |  |
| Hypertension,n(%) | No | 2653(65.4) | 2653(67.7) | 4.54 | 0.03 |
|  | Yes | 1402(34.6) | 1267(32.3) |  |  |
| Profession,n(%) | Agriculture | 3442(84.9) | 3274(83.5) | 29..38 | <0.01 |
|  | Animal husbandry | 120(3.0) | 87(2.2) |  |  |
|  | General staff | 264(6.5) | 226(5.8) |  |  |
|  | Housework or retirement | 229(5.6) | 333(8.5) |  |  |
| Education level,n(%) | Illiteracy | 1802(44.4) | 2234(57.0) | 153.54 | <0.01 |
|  | Primary school | 1251(30.9) | 973(24.8) |  |  |
|  | Junior high school | 682(16.8) | 462(11.8) |  |  |
|  | High school | 198(4.9) | 103(2.6) |  |  |
|  | College degree and above | 122(3.0) | 148(3.8) |  |  |
| Marital status,n(%) | Married | 3776(93.1) | 3460(88.3) | 81.37 | <0.01 |
|  | Unmarried | 82(2.0) | 63(1.6) |  |  |
|  | Live alone | 197(4.9) | 397(10.1) |  |  |
| Smoking,n(%) | Not smoke | 2868(70.7) | 3890(99.2) | 1253.59 | <0.01 |
|  | Smoking | 1187(29.3) | 30(0.8) |  |  |
| Drinking,n(%) | No alcohol | 3758(92.7) | 3907(99.7) | 260.87 | <0.01 |
|  | Drinking | 297(7.3) | 13(0.3) |  |  |
| Fatty Liver,n(%) | NO | 3240(79.9) | 3036(77.4) | 7.15 | <0.01 |
|  | YES | 815(20.1) | 884(22.6) |  |  |
| BPcate,n(%) | Normal blood pressure | 1148(28.3) | 1326(33.8) | 28.34 | <0.01 |
|  | Prehypertension | 1564(38.6) | 1393(35.5) |  |  |
|  | Hypertension | 1343(33.1) | 1201(30.6) |  |  |
| WCcate,n(%) | normal | 717(17.7) | 516(13.2) | 31.14 | <0.01 |
|  | obesity | 3338(82.3) | 3404(86.8) |  |  |
| WHRcate,n(%) | normal | 1418(35.0) | 1012(25.8) | 78.82 | <0.01 |
|  | obesity | 2637(65.0) | 2908(74.2) |  |  |
| HTC,n(%) | No | 2613(64.4) | 2623(66.9) | 5.41 | 0.02 |
|  | Yes | 1442(35.6) | 1297(33.1) |  |  |
| HLDL-C,n(%) | No | 3131(77.2) | 3110(79.3) | 5.28 | 0.02 |
|  | Yes | 924(22.8) | 810(20.7) |  |  |
| LHDL-C,n(%) | No | 1253(30.9) | 1533(39.1) | 59.06 | <0.01 |
|  | Yes | 2802(69.1) | 2387(60.9) |  |  |
| HTG,n(%) | No | 2813(69.4) | 3053(77.9) | 74.23 | <0.01 |
|  | Yes | 1242(30.6) | 867(22.1) |  |  |
| **Abbreviations:** continuous variables are expressed as mean ± standard deviation,Measurement data are expressed as n(%); ASCVD, atherosclerotic cardiovascular disease; WC, waist circumference; HC,hip circumference;FBG, fasting blood glucose; TG, triglycerides; TC, total cholesterol; HDL-C, high-density lipoprotein cholesterol; LDL-C, low-density lipoprotein cholesterol; SBP, systolic blood pressure; DBP, diastolic blood pressure; ALT, alanine aminotransferase; AST, aspartate aminotransferase; ALB, albumin; GLO, globulin; TBIL, total bilirubin; DBIL, direct bilirubin; ALP, alkaline phosphatase; GGT, γ-glutaminase; CR, creatinine; APOAB, apolipoprotein AB; APOB apolipoprotein B; HBDH,Hydroxybutyrate dehydrogenase; LDH,Lactate dehydrogenase;SD, pulse pressure difference ; WHR, waist-to-height ratio, BMI, body mass index; UA, uric acid; TyG, triglyceride blood glucose index; LAP, fat accumulation product index; BAI, body obesity index;VAI:Visceral Adiposity Index; LCI, blood lipid index; AI, arteriosclerosis index; AIP, plasma arteriosclerosis index; LpH, low-high-density lipoprotein ratio; THT, bilirubin comprehensive index,fhASCVD,family history atherosclerotic cardiovascular disease;fhDM,family history diabetes mellitus;DM,diabetes mellitus | | | | | |

| **Supplementary:Table 3.1** Screening variable subsets based on Cox regression in man | | | |
| --- | --- | --- | --- |
| Variable | Variable type | Variable | Variable type |
| LDH | Continuous | BPcate | Classification |
| UA | Continuous | DM | Classification |
| HDL-C | Continuous | Age | Continuous |
| SD | Continuous | DBP | Continuous |
| **Abbreviations**:LDH,Lactate dehydrogenase;UA, uric acid; HDL-C, high-density lipoprotein cholesterol;SD, pulse pressure difference ; DM,diabetes mellitus;DBP, diastolic blood pressure | | | |

| **Supplementary:Table 3.2** Screening variable subsets based on Lasso-Cox regression in man | | | |
| --- | --- | --- | --- |
| Variable | Variable type | Variable | Variable type |
| LDH | Continuous | Age | Continuous |
| UA | Continuous | Fatty liver | Classification |
| SBP | Continuous | DM | Continuous |
| HDL-C | Continuous | DBP | Continuous |
| **Abbreviations**:LDH,Lactate dehydrogenase;UA, uric acid; SBP, systolic blood pressure;HDL-C, high-density lipoprotein cholesterol; DM,diabetes mellitus; DBP, diastolic blood pressure | | | |

| **Supplementary:Table 3.3** Screening variable subsets based on RSF in man | | | |
| --- | --- | --- | --- |
| Variable | Variable type | Variable | Variable type |
| BPcate | Classification | AIP | Continuous |
| LHDL | Classification | BMI | Continuous |
| DM | Classification | FBG | Continuous |
| Smoking | Classification | BAI | Continuous |
| SD | Continuous | WC | Continuous |
| Hypertension | Classification | TyG | Continuous |
| DBP | Continuous | VAI | Continuous |
| UA | Continuous | WHR | Continuous |
| HC | Continuous | HDL-C | Continuous |
| SBP | Continuous | Age | Continuous |
| **Abbreviations**:DM,diabetes mellitus;SD, pulse pressure difference ;DBP, diastolic blood pressure;UA, uric acid;HC,hip circumference; SBP, systolic blood pressure; AIP, plasma arteriosclerosis index; AIP, plasma arteriosclerosis index;BMI, body mass index;FBG, fasting blood glucose; BAI, body obesity index;WC, waist circumference;TyG, triglyceride blood glucose index; VAI,Visceral Adiposity Index; WHR, waist-to-height ratio;HDL-C, high-density lipoprotein cholesterol | | | |

| **Supplementary:Table 3.4** Screening variable subsets based on Cox regression in woman | | | |
| --- | --- | --- | --- |
| Variable | Variable type | Variable | Variable type |
| LAP | Continuous | VAI | Continuous |
| SD | Continuous | LpH | Continuous |
| TBIL | Continuous | Fatty liver | Classification |
| HC | Continuous | HLDL | Classification |
| SBP | Continuous | DM | Classification |
| Age | Continuous | APOB | Continuous |
| FBG | Continuous | AI | Continuous |
| AIP | Continuous |  |  |
| **Abbreviations**:LAP, fat accumulation product index;SD, pulse pressure difference ;TBIL, total bilirubin;HC,hip circumference;SBP, systolic blood pressure;FBG, fasting blood glucose; AIP, plasma arteriosclerosis index;VAI,Visceral Adiposity Index; LpH, low-high-density lipoprotein ratio;DM,diabetes mellitus;APOB apolipoprotein B; AI, arteriosclerosis index | | | |

| **Supplementary:Table 3.5** Screening variable subsets based on Lasso-Cox regression in woman | | | |
| --- | --- | --- | --- |
| Variable | Variable type | Variable | Variable type |
| SBP | Continuous | FBG | Continuous |
| DBP | Continuous | AI | Continuous |
| HC | Continuous | LDL-C | Continuous |
| HDL-C | Continuous | Fatty liver | Classification |
| TBIL | Continuous | HLDL | Classification |
| VAI | Continuous | DM | Classification |
| Age | Continuous | APOB | Continuous |
| **Abbreviations**:SBP, systolic blood pressure;DBP, diastolic blood pressure;HC,hip circumference;HDL-C, high-density lipoprotein cholesterol;TBIL, total bilirubin;VAI,Visceral Adiposity Index;FBG, fasting blood glucose; AI, arteriosclerosis index;LDL-C, low-density lipoprotein cholesterol; DM,diabetes mellitus;APOB apolipoprotein B | | | |

| **Supplementary:Table 3.6** Screening variable subsets based on RSF in woman | | | |
| --- | --- | --- | --- |
| Variable | Variable type | Variable | Variable type |
| Fatty Liver | Classification | HC | Continuous |
| BPcate | Classification | BAI | Continuous |
| Hypertension | Classification | APOAB | Continuous |
| TG | Continuous | DBP | Continuous |
| LCI | Continuous | LDL-C | Continuous |
| LAP | Continuous | VAI | Continuous |
| AIP | Continuous | APOB | Continuous |
| SD | Continuous | WC | Continuous |
| SBP | Continuous | Age | Continuous |
| **Abbreviations**:TG, triglycerides; LCI, blood lipid index;LAP, fat accumulation product index;AIP, plasma arteriosclerosis index;SD, pulse pressure difference ;SBP, systolic blood pressure;HC,hip circumference;BAI, body obesity index;APOAB, apolipoprotein AB;DBP, diastolic blood pressure;LDL-C, low-density lipoprotein cholesterol;VAI,Visceral Adiposity Index;APOB apolipoprotein B;WC, waist circumference | | | |

| **Supplementary：Table 4. C statistic of different models on training and test sets** | | | | | |
| --- | --- | --- | --- | --- | --- |
| Model | Man | |  | Woman | |
|  | Training sets | Test sets |  | Training sets | Test sets |
| Cox regression | 0.760(0.726-0.793) | 0.779(0.736-0.822) |  | 0.774(0.752,0.795) | 0.733(0.697,0.768) |
| Lasso-Cox | 0.761(0.727-0.794) | 0.775(0.730,0.819) |  | 0.773(0.751,0.795) | 0.736(0.701,0.771) |
| RSF | 0.737(0.703-0.770) | 0.780(0.730,0.829) |  | 0.749(0.726,0.772) | 0.737(0.702,0.771) |
| China-PAR | 0.735(0.701-0.770) | 0.748(0.695,0.800) |  | 0.756(0.733,0.780) | 0.734(0.699,0.768) |
| FRS | 0.730(0.695-0.764) | 0.738(0.688,0.789) |  | 0.746(0.723,0.768) | 0.721(0.686,0.757) |
